# Critical Spatial-Temporal Dynamics and Prominent Shape Collapse of Calcium Waves Observed in Human hNT Astrocytes *in Vitro*

**DOI:** 10.3389/fphys.2022.808730

**Published:** 2022-06-17

**Authors:** Nicholas G. Mellor, E. Scott Graham, Charles P. Unsworth

**Affiliations:** ^1^ Department of Engineering Science, The University of Auckland, Auckland, New Zealand; ^2^ Department of Molecular Medicine and Pathology, School of Medical Sciences, The University of Auckland, Auckland, New Zealand; ^3^ Centre for Brain Research, The University of Auckland, Auckland, New Zealand

**Keywords:** *in vitro*, human neuroscience, astrocyte, criticality, shape collapse, calcium waves, intercellular dynamics, networks

## Abstract

Networks of neurons are typically studied in the field of Criticality. However, the study of astrocyte networks in the brain has been recently lauded to be of equal importance to that of the neural networks. To date criticality assessments have only been performed on networks astrocytes from healthy rats, and astrocytes from cultured dissociated resections of intractable epilepsy. This work, for the first time, presents studies of the critical dynamics and shape collapse of calcium waves observed in cultures of healthy human astrocyte networks *in vitro,* derived from the human hNT cell line. In this article, we demonstrate that avalanches of spontaneous calcium waves display strong critical dynamics, including power-laws in both the size and duration distributions. In addition, the temporal profiles of avalanches displayed self-similarity, leading to shape collapse of the temporal profiles. These findings are significant as they suggest that cultured networks of healthy human hNT astrocytes self-organize to a critical point, implying that healthy astrocytic networks operate at a critical point to process and transmit information. Furthermore, this work can serve as a point of reference to which other astrocyte criticality studies can be compared.

## Introduction

Astrocytes are glial cells found in the central nervous system and were initially thought to be primarily a passive cell responsible for the physical and chemical support of neurons (Allen and Barres, 2009). However, contemporary work, has highlighted that astrocytes are far from being perfunctory, rather they can respond to a host of stimuli, including neurotransmitters, from multiple sources such as neurons (Cornell-Bell and Fink, 1990; Scemes and Giaume, 2006). While neurons respond to stimuli through action potentials, astrocytes are electrically inert. Instead, astrocytes display calcium (Ca^2+^) based excitability (Cornell-Bell and Fink, 1990). Whereby, stimuli are transduced into elevations in internal Ca^2+^ concentrations, commonly known as Ca^2+^ waves (Zorec et al., 2012). Bidirectional communication between astrocytes and neurons is made possible by the astrocytes ability to release neurotransmitters such as ATP and glutamate (Allen and Barres, 2005; Volterra and Meldolesi, 2005; Scemes and Giaume, 2006). Astrocytes have also been observed to strategically localise in close proximity to neuronal synapses, giving rise to the discovery of the tripartite synapse (Perea et al., 2009). Through the release of active substances and their physical placement in the brain, astrocytes are thus thought to play a role in potentiation of neuronal synapses (Allen and Barres, 2005). In addition to neuronal and astrocytic bidirectional signalling astrocytes form independent networks with the ability for long distance communication. This communication occurs through gap junction mediated diffusion of molecules such as inositol-3-phosphate, or extracellular release and diffusion of neurotransmitters (Verkhratsky and Kettenmann, 1996; Giaume and Naus, 2013). These two pathways of communication have the ability to elicit Ca^2+^ waves in the receiving astrocyte, leading to regenerative Ca^2+^ waves spreading over large distances in the network. Thus, astrocytic networks can be viewed as a network of dynamic elements where signalling and information propagation occurs.

Criticality is a property exhibited in dynamic networks. Large systems of interacting dynamic elements often do not display events predicted by the analysis of each individual element. Thus, criticality is used to link single element events to network wide events, by proposing that it is the same identical generative mechanisms that cause events at all scales (Bak and Chen, 1991). Events are thus scale free, a property that results in a power-law distribution of event sizes (Bak et al., 1987). Various dynamical systems have been analysed using this method, including but not limited to the sizes and frequencies of earthquakes (Leet et al., 1950) and forest fires (Drossel and Schwabl, 1992), magnetic particle spins within magnetic materials, in both empirical experiments and simulations (Sethna et al., 2001), swarm sizes in groups of insects (Attanasi et al., 2014), stock market fluctuations (Bouchaud, 2000), and failures in power grids (Carreras et al., 2000). Systems such as magnetic particles require a parameter to be tuned so that the system undergoes a phase transition from a subcritical or supercritical regime to a critical state. However, other models, such as the ‘sandpile’ model, were developed to omit a tuning parameter and instead “self-organise” to a critical point (Bak et al., 1987). Self-organising models led to the *hypothesis* that the brain, which does not have a tuneable parameter, is a network of neurons that are organised to a critical state. This led to criticality research expanding to neural networks *in vitro* using microelectrodes on cultured neurons (Beggs and Plenz, 2003; Friedman et al., 2012), and later the human brain, using functional MRI (Kitzbichler et al., 2009), non-invasive (Thatcher et al., 2009) as well as invasive electroencephalogram (EEG) (Meisel et al., 2012). Critical dynamics were also identified in macaque monkeys using chronically implanted microelectrode arrays (Petermann et al., 2009). Later studies have looked at larger regions of the brain using genetically encoded indicators in mice cortex (Scott et al., 2014) and more recently in the whole brains of zebra fish (Ponce-Alvarez et al., 2018). Non-invasive human studies using MEG have also identified aspects of critical systems in the human brain at rest and during tasks, suggesting these types of dynamics arise through the network structures not through exogenous activity (Matias et al., 2013; Shriki et al., 2013).

Networks of neurons are typically studied in the field of Criticality. However, the study of astrocyte networks in the brain has been recently lauded to be of equal importance to that of the neural networks (Giaume et al., 2010). The most extensive study of criticality in networks of astrocytes was performed by Jung *et al* in 1998 and 2001 (Jung et al., 1998, 2001). In this work astrocyte cultures, from healthy rats, and neurological resections of intractable epilepsy in human patients, were analysed (Jung et al., 2001). Imaging was performed by loading the astrocyte cultures with a calcium, Ca^2+^, sensitive dye, and then recording images. Using these images clusters of Ca^2+^ waves were identified, and the size of these clusters measured. It was found that the distributions of these sizes followed a power-law (Jung et al., 1998, 2001). Contemporary work has examined Ca^2+^ waves inside single astrocyte cells using a similar method and found that sizes of single cell clusters of Ca^2+^ waves also follow a power-law distribution (Wu et al., 2014).

A potential reason for the lack of criticality studies in networks of astrocytes is that neurons are electrical cells, and thus their electrical activity is measured via electrodes which record action potentials. Experiments with neurons enjoy the luxury of high sampling rates and can be performed over long term experiments providing vast quantities of multi-channel data that is ideal for criticality analyses. Astrocytes, however, are non-electrical cells whose main communication pathway instead is via Ca^2+^ waves that carry information between cells, and so recordings must be made through live cell imaging of Ca^2+^ using fluorescent imaging modalities. While electrode recordings are conducive to long term experiments, live cell imaging is not, due to dye photo-bleaching and is metabolized out of the cell a process which takes around 1 h, thus limiting the imaging period. Photo-toxicity is another limiting factor which results in damage to cells after extensive exposure to light during the imaging process (Laissue et al., 2017).

Our group is involved in understanding the calcium communication in networks of human hNT astrocytes (Raos et al., 2013, 2017; Jordan et al., 2016; Li et al., 2019). Astrocytes were commonly thought of as perfunctory cells, however, recent evidence has demonstrated that they possess far more functionality and complexity than was commonly thought. Thus, our motivation, is to examine if networks of human astrocytes using criticality to further demonstrate the complex functionality of such cells in networks. To achieve this we examine the criticality of astrocytes differentiated from the human embryonal carcinoma derived NTERA2/D1 (hNT) cell line (Pleasure et al., 1992). hNT derived astrocytes are a robust and reliable model that have been shown to be a valid alternative to primary human astrocytes (Haile et al., 2014), as well as showing potential in transplant therapy post-stroke (Hara et al., 2008), and displaying a functional neuron astrocyte lactate shuttle system (Tarczyluk et al., 2013).

## Materials and Methods

In this section the methods used to assess criticality are explained. Firstly Ca^2+^ imaging was performed, the images were then converted to time series, then avalanches were found in the time-series, and finally shape collapse was assessed in the avalanches.

### Cell Culture, Cell Labelling and Imaging

hNT astrocytes were grown according to previously described protocols (Pleasure et al., 1992; Unsworth et al., 2011). Initially Human hNT precursor cells were cultured in a petri dish in DMEM:F-12 (Gibco, Cat# 11330032) supplemented with 10% fetal bovine serum (FBS) and 10 µm retinoic acid. The cells were replated with fresh media every 2–3 days for 14 days. They were then transferred to T-75 flasks and the media changed every 2–3 days for 10 days. At this point cells with neural morphology were harvested via select trypsinization. The remaining cells were replated into a T-75 flask and cultured with DMEM:F-12 supplemented with 5% FBS, and the mitotic inhibitors uridine (Urd), 5-fluoro-2′-deoxyuridine (FUdR) and β-D-arabinofuranoside (AraC) for 5 weeks Urd at a concentration of 10 μM was applied to the precursor cells from day 31 until astrocytes were harvested on day 61. FUdR was applied from day 31 until day 55, initially at a concentration of 10 μM between days 31 and 41 and then at 5 μM from day 41 until day 55. AraC was applied at a concentration of 1 μM between days 31 and 41. On day 61, hNT astrocytes were harvested with 0.05% trypsin. All cell incubation was performed at 37°C and 5% CO_2_.

In order to image the astrocytes they were seeded onto 35 mm Petri dishes. This was done by first adhering a small hollow cylindrical PDMS structure, area of approximately 50 mm^2^ into the middle of the Petri dish to reduce the seeding area. 200 µL of cell suspension was then added to the PDMS structure. The cell suspension was produced by centrifuging astrocytes in a falcon tube at 300 × *g*, removing the supernatant, and adding 1 ml DMEM:F-12 to the Falcon tube. Cell density in this suspension was then calculated by cell counting, the density was the diluted to the required density for seeding. The seeding density was 20,000 cells per PDMS structure, the same as the astrocyte density upon astrocyte harvest. These were then incubated overnight to allow the cells to adhere. The following day the PDMS structures were carefully removed and the petri dishes filled with 2.5 ml of media, and then incubated overnight and imaged the next day.

To enable live cell imaging of Ca^2+^, astrocytes were incubated with 1 µm Fluo-4 AM (Invitrogen, Cat#F14201) dye for 30 min in the cell culture incubator. The astrocytes were then rinsed with FluoroBrite (Gibco, Cat#A1896701) supplemented with 5% FBS twice. 6 ml of the supplemented FluoroBrite media was then added to Petri dishes which were then placed in the incubator for 15 min to allow the astrocytes to settle.

Petri dishes were then transferred to the microscope incubator and imaged using an Olympus BX53 upright microscope at 10× magnification. Recordings were made for 40 min with 4 × 4 pixel binning. In order to reduce photo bleaching an image was captured every 2 s, each image had an exposure time of 0.640 s. For the remaining 1.36 s the illumination shutter was closed. The resulting image stack was 480 × 640 pixels with 1201 images.

### Image and Signal Preprocessing

Astrocytes were identified using a maximum intensity z projection of each image stack, this produces an image where each pixel contains the maximum value over all images in the stack for each pixel location. From this image astrocytes were manually labelled as a region of interest (ROI). The coordinates of each ROI determined, and the mean pixel intensity for each ROI in all images in the stack was calculated, giving a time-series that has 1201 columns and the number of rows equal to the number of ROIs. This time-series recorded information on changes in Ca^2+^ within the imaged astrocytes.

The ROI coordinates and time-series were processed and analysed using the Matlab^©^ computing environment. The time-series were filtered, normalized and baseline corrected using the method of (Jia et al., 2011). In order to perform avalanche analysis, the time-series was first made binary. This was performed by estimating the noise in each ROI time series and thresholding the signal at a value of $3\sigma_{noise}$ , where $\sigma_{noise}$ is the standard deviation on the estimated noise (Romano et al., 2017).

### Avalanche Analysis

Avalanche analysis was performed using a similar method to the spatiotemporal cube method which was first used by (Jung et al., 1998) in astrocyte networks. This method works by stacking frames and when an astrocyte is active, namely binary high in its time-series, its ROI in the corresponding frame reflects this. The result is similar to the captured image stack but the fluorescent information has now been made binary. ROIs used in the spatiotemporal cube were also dilated so that neighbouring astrocytes overlapped. Dilation was performed using a circular structuring element with a radius equal to the average distance to the nearest six neighbouring cells, this is similar to recent methods which produced a square around each cell with side length equal to the average distance to the nearest six cells (Gosak et al., 2017).

Within the spatiotemporal cube, there could exist overlapping active cells, both spatially and temporally. It was these overlapping cells that were then joined together to form a volume within the spatiotemporal cube, this volume was defined as an avalanche (Jung et al., 1998; Gosak et al., 2017). The temporal size of the avalanche was defined as the number of active cells that spatially overlapped at each time point. An avalanche was defined to end when there were no active cells that could be joined to the volume in the subsequent temporal frame. If there were two or more avalanches which were separate but then collided these were combined into a single avalanche, as has been done in avalanche analysis of astrocytes (Jung et al., 2001) and beta cells (Gosak et al., 2017). Avalanche size was then defined as the sum of the temporal size (i.e. the number of cell active in an avalanche at each distinct time point within that avalanche) over the duration of the avalanche. The duration was defined as the time between the initiation and end of an avalanche.

### Power-Law Fitting

Both the size and duration distributions were fit using a ‘maximum likelihood estimation’ (MLE) based method. MLE gives better fits to power-law distributed data than least squares fits and is widely used in this type of analysis (Goldstein et al., 2004; Bauke, 2007; Clauset et al., 2009; Klaus et al., 2011).

The generic power-law probability density function (PDF) was given by Eq. 1.

$$p\left(x\right)=Cx^{-\alpha }$$ (1)

Where, C was a normalization constant so that $P\left(x\right)=\;\overset{\infty }{\underset{-\infty }{\int }}p\left(x\right)=1$ . As size and duration can never be negative x>0 . Additionally, the pdf diverged as x→0 so there existed a lower bound, $x\geq x_{min}$ , to this model. There can also be an upper bound $x\leq x_{max}$ . This upper bound could be either the experiment duration, or the size of the network being studied. Knowing these bounds allowed for the determination of C , which was given by Eq. 2.

$$C=\frac{\alpha -1}{x_{\text{min}}^{1-\alpha }-x_{max}^{1-\alpha }}$$ (2)

The estimation of $x_{min}$ follows a similar method to (Clauset et al., 2009; Deluca and Corral, 2013) where the distribution data was truncated for different values of $x_{min}$ . Each truncated data set was then fit with the power-law distribution model using the MLE and the Kuiper’s statistic was calculated.

Next, goodness-of-fit was assessed using a method put forward by Lilliefors for exponential and normal distributions (Lilliefors, 1967, 1969) which was later adapted to the power-law distribution by Goldstein *et al* (Goldstein et al., 2004), and has been widely used (Clauset et al., 2009; Deluca and Corral, 2013; Alstott et al., 2014). This involved first fitting the power-law model and calculating the Kuiper’s statistic for that fit. Using the parameter estimated from the power-law model a large number of synthetic power-law distributions were generated. These synthetic distributions were then fit and the Kuiper’s statistic for each distribution was calculated. The fraction of synthetic fits with a Kuiper’s statistic greater than the Kuiper’s statistic from the empirical data produces a *p*-value which was a measure of how good the power-law fit was. The critical *p*-value used in this analysis was $p_{c}$ = 0.1, a fit with a *p*-value less that $p_{c}$ was deemed to fail the goodness-of-fit test. The power law was fitted across all the data greater than $x_{min}$ and all the scaling regions for the following reasons. The number of datapoints that exist in the lower scaling region (low duration and size) was found to be sufficient to provide a good approximation to the power-law. Whereas the number of datapoints in the higher scaling region (high duration and size) was fewer making for a poorer approximation of the data to a power-law. Thus, by maximising the data used and fitting across the whole distribution would help to minimise any error in the system.

Finally, from the power-law fits that passed the goodness-of-fit test the smallest $x_{min}$ was selected. A similar method, was then used to find $x_{max}$ , however $x_{max}$ was found to have less impact on the MLE estimation of α , and so $x_{max}$ was set to the largest observed value in the distribution. Simulated data was generated using the transformation method (Press et al., 2007).

An estimate in the uncertainty in the lower bound of the scaling region can be calculated. This was performed through bootstrapping by drawing a uniform random sample of *n* points from the original data, fitting this using the previously described methods, and finally estimating $x_{min}$ and calculating the standard deviation in the estimate of $x_{min}$ across a number of bootstrapping repetitions (in this work 1000 repetitions was used) (Clauset et al., 2009).

### Model Comparison

Along with the power-law model there were other heavy tailed distributions that could be used to fit the avalanche data. Alternate models assessed were the lognormal, gamma, exponent, and generalized pareto distributions. Each of the distributions were fit to the data then compared to the power-law model and the model with the most evidence in its favour was selected as the model, Akaike information criterion (AIC) was used for this selection process (Akaike, 1973). A finite sample size corrected variant of AIC was used, called AIC_c_. This formulation adjusts for the ratio of sample size to the number of model parameter (Burnham and Anderson, 2002). The equations for AIC and AIC_c_ are given in Eqs 3, 4 respectively. Where, $L\left(\hat{\theta }\right)$ is the maximum likelihood estimation of a particular model, K is the number parameters in the model, and n is the sample size used to fit the model.

$$AIC=\;-2\log\left(L\left(\hat{\theta }\right)\right)+2K\;$$ (3)

$$AIC_{c}=-2\log\left(L\left(\hat{\theta }\right)\right)+2K+\frac{2K\left(K+1\right)}{n-K-1}\;$$ (4)

From the calculation of a set of AIC_c_ values the AIC_c_ differences can be calculated as given by Eq. 5.

$$\Delta_{i}=\;AIC_{c_{i}}-AIC_{c_{min}}\;$$ (5)

Here, $\Delta_{i}$ was a measure of the empirical support for model *i*, and AIC_cmin_ was the smallest AIC_c_ value from the set of candidate models. From the $\Delta_{i}$ values Akaike weights, $w_{i}$ , could be calculated as given by Eq. 6.

$$w_{i}=\frac{\exp\left(-\frac{1}{2}\Delta_{i}\right)}{\sum_{r=1}^{R}\exp\left(-\frac{1}{2}\Delta_{r}\right)}$$ (6)

These weights could be viewed as the weight of evidence in favour of a model being the actual model given one of the models in a set of models must be the best model (Burnham and Anderson, 2002; Wagenmakers and Farrell, 2004). There are limitations of this type of model comparison. The first was that it measures which model in a set of models was the most likely to produce the data, and so all models which may produce the data must be in that set, additionally models must be fit to the same data set.

### Shape Collapse

In order to assess shape collapse in avalanche temporal profiles the average shape for each duration,  S(T) , was calculated. Each average temporal profile was then normalized using the maximum value of the temporal profiles and the standard deviation of each individual profile. From these normalized profiles the variance was the variable that was minimized by Eq. 7.

$$\text{Min}f\left(a\right),\;where\;f\left(a\right)=var\left(\langle S_{N}\rangle \left(\frac{t}{T}\right)T^{-\left(b-1\right)}\right)$$ (7)

Where, $S_{N}\left(\frac{t}{T}\right)$ was the average normalized temporal profile of avalanches of duration T , normalized to duration 1, and *b* is the shape collapse exponent. This method produced similar results to the method which uses the span of the average temporal profiles to the normalize (Marshall et al., 2016). In order for a temporal profile to be considered in the shape collapse it needed have T>3 , as well as there being at least three realizations of the duration. Previously, temporal profiles with fewer than 20 realizations were removed, however, due to the type of data recorded from astrocytes this was not possible and so a lower threshold was used (Marshall et al., 2016).

Additionally, the distribution of $\overset{T}{\underset{0}{\int }}S\left(T\right)dT$ was calculated resulting in the average size given a duration distribution, which according to criticality theory should obey a power-law $\overset{T}{\underset{0}{\int }}S\left(T\right)dT\;∼\;T^{\frac{1}{\sigma \nu z}}$ . As this was not a probability distribution but a scaling relationship the MLE method cannot be used and instead a least squares fit was used to estimate the exponent.

## Results

The avalanche data recorded resulted in two data distributions, duration and size, for each of the six analysed networks. In order to analyse if astrocyte networks exhibited criticality they were first fit with multiple competing models to see if the evidence suggested a power-law model was the most likely model. Next the critical exponents from the power-law models were calculated. Finally, another aspect of criticality, shape collapse was assessed in the temporal profiles of the avalanches.

### Viability of Networks

**FIGURE 1:**
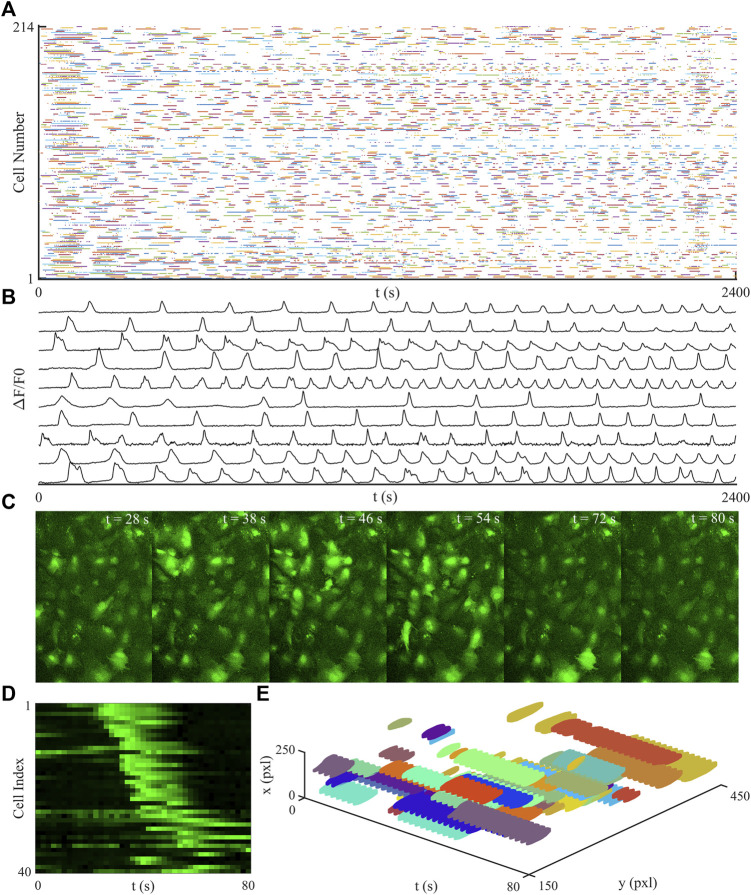
Plots of a typical time-series recording. **(A)** shows a raster plot of a typical network over the full recording period. **(B)** is a time-series of a typical cells showing Ca^2+^ waves, there are regular transients of different shapes and frequencies displayed. **(C)** is an image sequence showing a typical avalanche, the avalanche is initiated in a single cell with the Ca^2+^ wave spreading and activating further afield cells. The avalanche ends when the initial cells become inactive and the wave no longer spreads. This same avalanche is displayed as a heatmap in **(D)**, where cell index relates to cells sorted by distance from the initial point of the avalanche, and as a 3D plot in **(E)**.

To assess the viability of the imaged networks the number of cells and the number of active cells which showed a Ca^2+^ transient were counted. On average there was 254 ± 42 cells per network, of these cells the majority displayed Ca^2+^ transients, with the maximum number of inactive cells in one network being 7, an inactive cell was defined as a cell which did not display any Ca^2+^ waves. Shown in Figure 1 is a raster plot of a network, Figure 1A, as well as a time series of showing regular Ca^2+^ waves from multiple cells, Figure 1B. Figure 1C displays an avalanche as an image sequence, as well as a heatmap, Figure 1D. This same avalanche is displayed in Figure 1E as a 3D plot. This same avalanche is displayed in Figure 1E as a 3D plot. A typical avalanche starts when one or more cells become active, this produces a Ca^2+^ wave, which is then transmitted to the nearest neighbours of the initiating cell. Further afield cells are then also activated with the avalanche front propagating away from the initiating cell. The avalanche ends when the initiating cell, and then its neighbours, become deactivated and the propagating front dies out.

### Distribution of Best Fit

For all six data sets, both duration and size, a scaling range was identified within which the evidence was in favour of the data being drawn from a power-law distribution, given that one of the considered models was the AIC_C_ best model. The lower bound on the duration data scaling range across all data sets was found to be 21.4 ± 6.6 s, for the size data this was 10.3 ± 6.8 cells. Below this lower bound the model with the most AIC_C_ evidence was the lognormal for both duration and size distributions and all data sets. At and above the lower bound of the power-law range the AIC_C_ evidence for a power-law was high, as most data sets had $w_{1}\gg w_{2}$ (where $w_{1}$ corresponded to the model with the largest Akaike weight). Network 6 duration distribution was found to have $w_{1}$ = 0.613 (corresponding to the power-law), and $w_{2}$ = 0.387. While the difference in Akaike weights was closer than other networks, the power-law model was found to be 0.613/0.387 ≈ 1.6 times more likely in terms of its AIC_C_ discrepancy than the second most likely model, a lognormal model in this example (Wagenmakers and Farrell, 2004). Additionally, increasing the minimum duration by 2 s above this lower bound resulted in the evidence for a power-law becoming much higher with $w_{1}$ ≈ 1.

**FIGURE 2:**
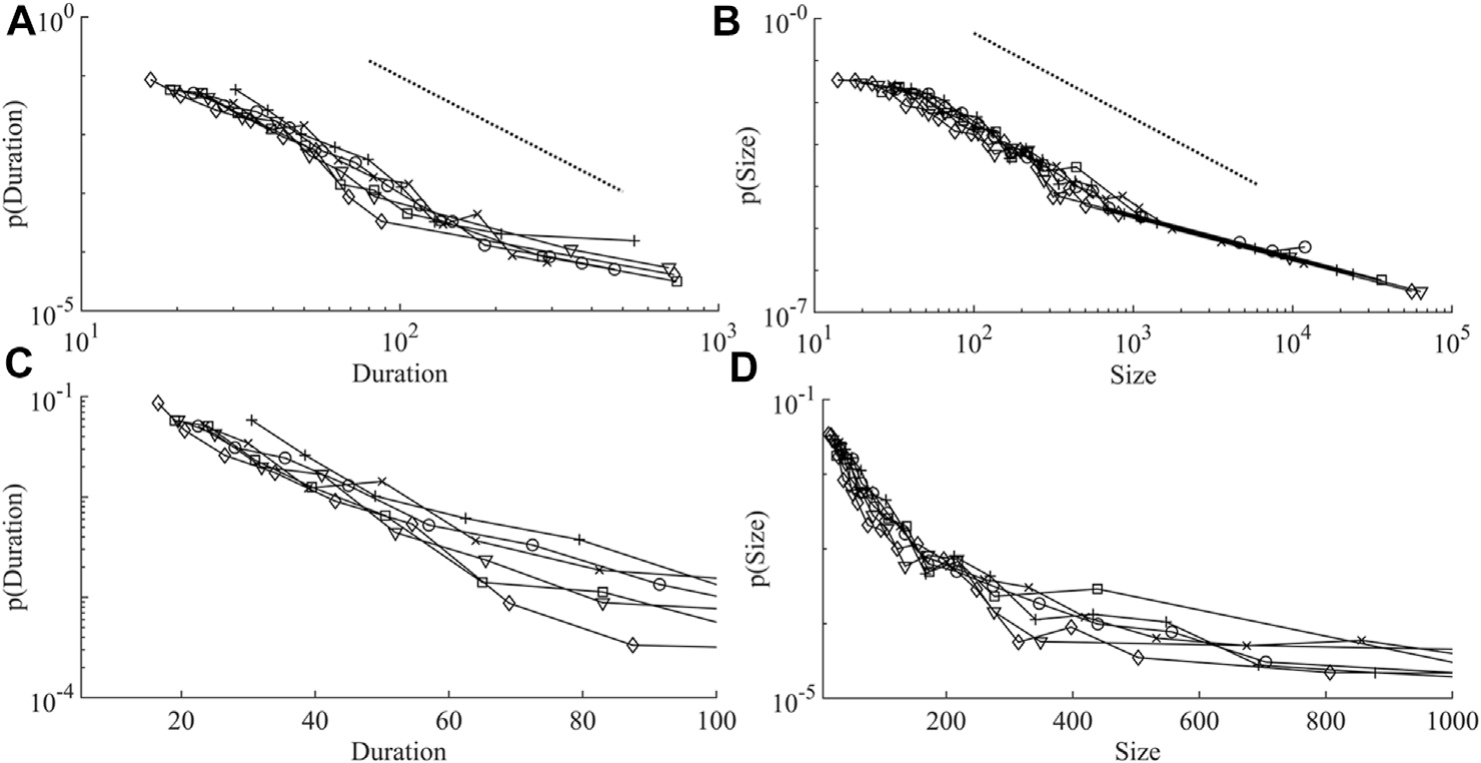
Duration and size distributions in all networks. **(A)** shows the duration distributions, the dashed line is a perfect power-law with exponent 2.8. **(B)** shows the size distributions, the dashed line is a perfect power-law with exponent 2.06. **(C,D)** are semilog (log y, linear x) plots of the initial parts of both distributions. Network 1 (o), network 2 (x), network 3 (+), network 4 (□), network 5 (▽), and network 6 (◇).

**FIGURE 3:**
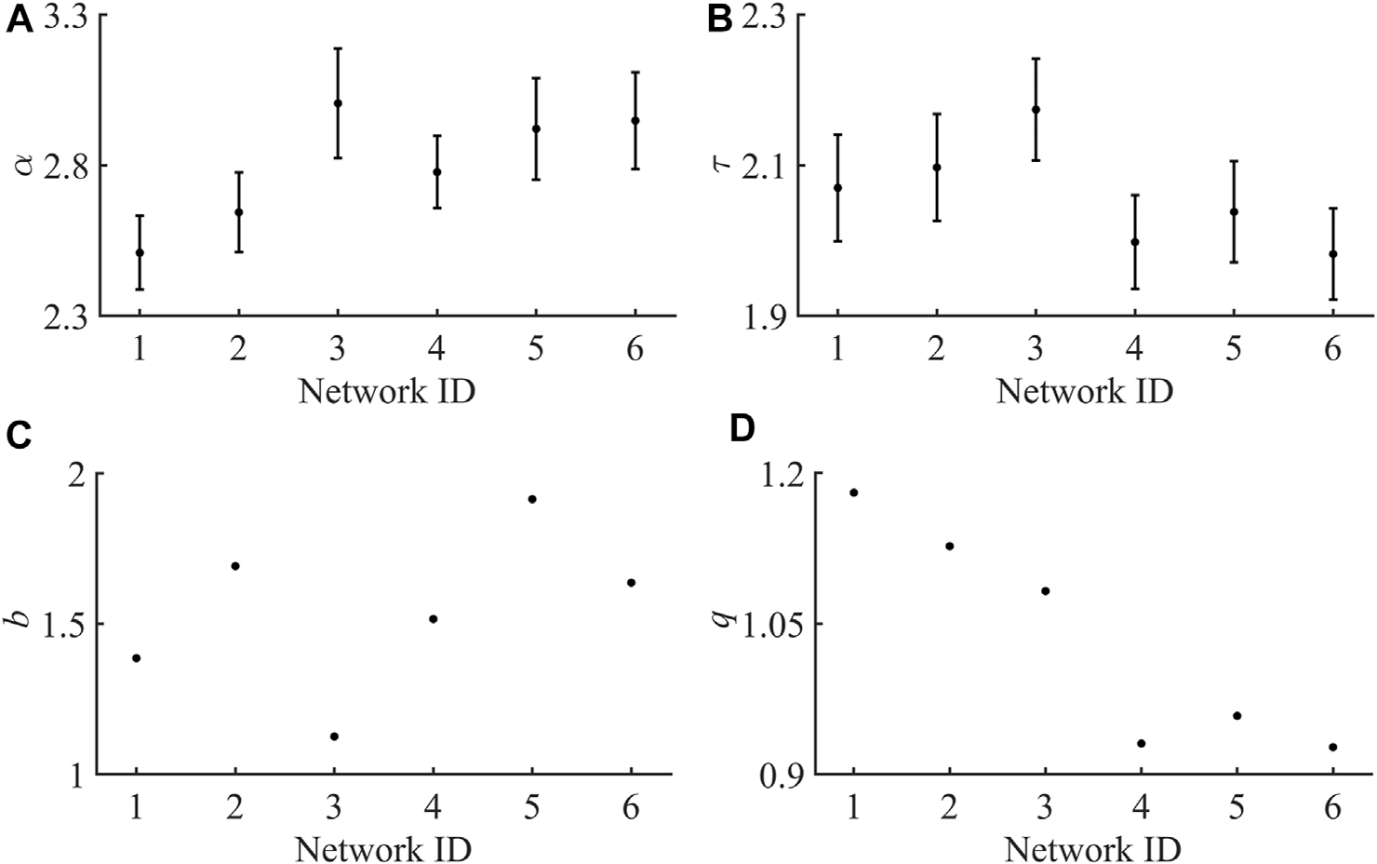
Plots for each individual exponent across all six hNT astrocyte networks. **(A)** Duration distribution exponents ( α ). **(B)** Size distribution exponent ( τ ). **(C)** Shape collapse exponent **(B)**. **(D)** The Scaling relation $\left(q=\frac{\mathrm{\sigma \nu z}\left(\alpha -1\right)}{\left(\tau -1\right)}\right)$ . (Error bars represent the standard deviation in the estimate).

Although, at this lower bound, the best considered model was the power-law the fit of the model was not satisfactory according to the goodness-of-fit test so a smaller scaling region needed to be considered. Within this smaller scaling region there are multiple fits and one needed to be chosen according to a set of criteria. The chosen criteria was to select the model that was fit to the smallest $x_{\text{min}}$ (largest number of data points), given that $p>p_{c}$ ( $p_{c}$ = 0.1). Other criteria could be used, such as the smallest Kuiper’s statistic, or a combination of Kuiper’s statistic and the number of data points. These were explored and were found not to affect the results in a significant manner. It was found that of both the duration and size distributions across the six networks, all duration and size distributions produced p values larger than $p_{c}$ . The models which passed the goodness-of-fit test were analysed and the lower bound for the duration data was 39.3 ± 8.4 s, and 24.5 ± 8.2 cells for the size data. The average number of avalanches that were fitted in the duration data was 186 ± 43, and 271 ± 30 for the size data. The final duration and size distributions that were fit are shown in Figures 2A,B respectively. These appear as linear lines in a log-log plot, which is expected from a power-law. Two distinct scaling regions might exist in the data, as can be observed in Figure 3. However, the number of datapoints that exist in the lower scaling region (low duration and size) is sufficient for a good approximation to be made to the power-law. Whereas the number of datapoints in the higher scaling region (high duration and size) is fewer making for a poorer approximation of the data to a power-law.

Figure 2, shows that most networks display one large avalanche that corresponds to that networks longest avalanche. Hence, for the largest avalanche, with a size of around 70,000 and duration of 1560 s, on average only 45 cells must be active at each time point, far below the average network size. This was observed by (Jung et al., 2001) in their intracellular calcium wave model of astrocytes: “There is one dominating huge cluster that forms the backbone of the temporal evolution of the array. Such a mother cluster is typical for systems above propagation threshold and small to moderate noise.” (Jung et al., 2001).

### Power-Law Exponents

Table 1 shows the scaling exponents of the best fit power-law model. Averaging across all networks it was found that for the duration distributions α = 2.80 ± 0.19 where $P\left(T\right)∼T^{-\alpha }$ , and for the size distributions τ = 2.06 ± 0.07, where $P\left(S\right)∼S^{-\tau }$ . Figure 2, shows the mean power-law fit to both the duration and size distribution data sets.

**TABLE 1:** Table of exponents calculated from analysed networks.

ID	α	$T_{min\;}$	$T_{max\;}$	$N_{\alpha }$	τ	${\text{S}}_{\text{min }}$	${\text{S}}_{\text{max }}$	$N_{\tau }$	$\frac{1}{\sigma \nu z}$	q	*b*	DCC	NMSE
1	2.51 ± 0.12	40 ± 12 s	1052 s	223	2.07 ± 0.07	29 ± 10	13363	248	1.66	1.18	1.39	0.25	0.43
2	2.64 ± 0.13	42 ± 16 s	650 s	215	2.10 ± 0.07	27 ± 8	13144	265	1.69	1.13	1.69	0.19	0.52
3	3.01 ± 0.18	54 ± 6 s	1216 s	151	2.17 ± 0.07	36 ± 14	26757	329	1.85	1.08	1.13	0.14	0.26
4	2.78 ± 0.12	34 ± 8 s	1658 s	237	2.00 ± 0.06	24 ± 14	40365	269	1.66	0.93	1.52	0.12	0.37
5	2.92 ± 0.17	36 ± 9 s	1560 s	139	2.04 ± 0.07	18 ± 7	71297	245	1.77	0.96	1.91	0.08	0.23
6	2.95 ± 0.16	30 ± 7 s	1622 s	152	1.98 ± 0.06	13 ± 6	62584	270	1.84	0.93	1.64	0.14	0.32
mean	2.80	39.3 s	1293 s	186	2.06	24.5	37918	271	1.75	1.03	1.54	0.16	0.36
$\sigma_{\text{mean}}$	0.19	8.4 s	397 s	43	0.07	8.2	24778	30	0.09	0.11	0.27	0.06	0.11
3D Ising (Perković et al., 1995)	2.81 ± 0.11	N.A.	N.A.	N.A.	2.03 ± 0.03	N.A.	N.A.	N.A.	1.75	1	1.75*	N.A.	N.A.

The other exponent of interest from the duration and size data is the exponent from the average size of a given duration data, $S\left(T\right)∼T^{\frac{1}{\sigma \nu z}}$ . Across all networks the average value was found to be $\frac{1}{\sigma \nu z}$ = 1.75 ± 0.09. Additionally, the scaling relation $\frac{\alpha -1}{\tau -1}=\frac{1}{\sigma \nu z}$ should be obeyed when a system is critical. Thus, an additional value could be defined as $q=\frac{\sigma \nu z\left(\alpha -1\right)}{\left(\tau -1\right)}$ , which if the scaling relations were correct would be equal to unity (Ponce-Alvarez et al., 2018). Across all networks the mean value of q  = 1.03 ± 0.11, and networks 1 and 2 were found to have values of q the most different from unity. Figure 3 is a plot of the exponents from all six networks. Mean exponent values for both the size (τ) and duration (α) distributions, as well as the size given duration exponent (1σvz) are closely approximate to those of a simulated 3D Isiing model subjected to an external driving force (Perković et al., 1995). Thus, the universality class of an astrocyte network is approximated by the 3D Isiing model of Perkovic *et al* (Perković et al., 1995). In the table N.A. means the value is not applicable to model and * means that the value was not available in (Perković et al., 1995) but is the theoretical value.

The Deviation from the Criticality Co-efficient (DCC) of (Ma et al., 2019) is defined as:

$$\text{DCC}\;=\;\left|\frac{\alpha -1}{\tau -1}-\frac{1}{\sigma \nu z}\right|$$ (8)

It was observed that the mean DCC was below the threshold = 0.2 of Ma *et al*, with only one network existing above this threshold. DCC values for all networks are presented in Table 1 **.**

### Shape Collapse of Avalanches

Another aspect of critical systems is that the temporal profile of avalanches will, under the correct renormalization, collapse down to a single universal scaling function. This is shown mathematically as $S\left(t,T\right)=T^{b-1}S\left(t/T\right)$ , where S(t/T) is the universal scaling function (Sethna et al., 2001). Additionally, this scaling exponent is related to the exponent of the average size of a given duration distribution, $b=\frac{1}{\sigma \nu z}$ , and so a system at criticality should obey this relationship (Sethna et al., 2001; Friedman et al., 2012).

**FIGURE 4:**
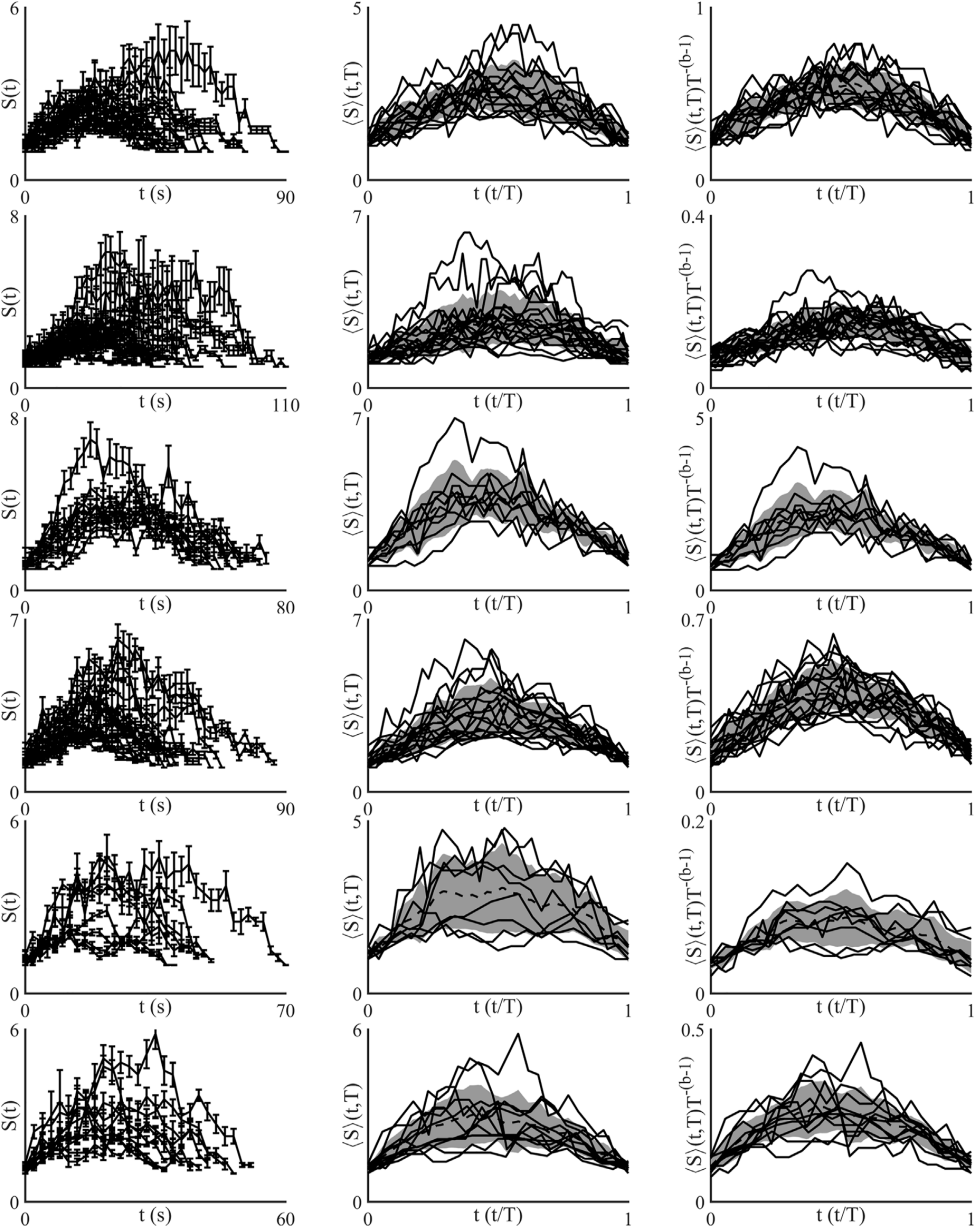
Shape collapse of all networks analysed. The top row of images corresponds to network 1, with the subsequent rows corresponding to subsequent networks. All networks show a universal shape similar to a parabola, although the shape has a slight left skew. Network 3, third row, shows the poorest shape collapse.

**FIGURE 5:**
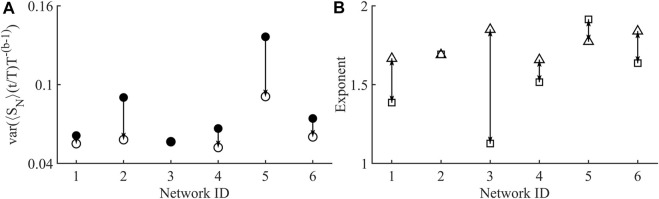
**(A)** Plot showing the variance of the avalanche temporal profiles before (•), and after collapse (o). Single arrow shows how the behaviour before collapse does actually tend to lower values after collapse which is to be expected in a critical system (Sethna et al., 2001). Network 3, shows a very small decrease in variance after collapse, although its starting variance is low compared to the other networks. **(B)** Plot showing distance between the shape collapse exponent **(B)** (□) and size given duration exponent $\left(\frac{1}{\mathrm{\sigma \nu z}}\right)$ (△). Double arrows highlight the closeness in proximity that exists in these real-world astrocyte networks which is expected in an ideal critical system to be equal. Network 2, shows very close agreement between the two exponents.

The shape collapse of all networks is shown in Figure 4. All networks show a similar universal shape, close to that of a parabola. Shape collapse can also fail, which means there was no exponent within the examined range that collapsed the avalanches, this did not occur in any of the examined networks. Shape collapse reduced normalised variance in all networks excluding network 3, which produced a shape collapse exponent of 1.13, but showed almost no change in normalised variance, Figure 5A. Currently there is no widely used metric for assessing the quality of shape collapse. One metric suggested is the normalized mean squared error (NMSE) of height-normalized individual profiles to the combined normalized average of all collapsed profiles as defined in (Miller et al., 2019). All of the collapsed profiles were below the threshold of NMSE = 1 given in Miller *et al.* Shown in Figure 5B is the agreement between the size given duration and shape collapse exponents, in an ideal system these are equal.

## Discussion

This research has shown that cultures of human hNT astrocytes do exhibit aspects of criticality. In order to assess if these cells displayed criticality they were grown into networks and the Ca^2+^ waves they displayed were recorded. Avalanche analysis was then applied in a similar way to the methods used to assess criticality in recordings of neurons.

Many recent studies have found power-law scaling of avalanche size and duration distributions, one aspect of criticality, in biological recordings. However, questions have been raised as to whether the data purported to being power-law does indeed have statistical support necessary to make this claim, or if another heavy tailed distribution, e.g. lognormal, fit the data better (Clauset et al., 2009; Stumpf and Porter, 2012). All networks assessed here showed scaling regions that were, according to AIC, most likely generated by a power-law process.

It should be noted that this study is not involved with the measurement of the criticality of neurons but rather astrocytes where the recording modalities are also different. In the only two criticality articles on astrocytes, exponential cut-offs were not reported (Jung et al., 1998; Wu et al., 2014). Similarly, in this human astrocyte work exponential cut-offs were not observed. This could imply three things either the imaging modality that can only be used to observe the Ca^2+^ communication in astrocytes does not permit cut-offs to be observed or in fact astrocyte behaviour does not exhibit cut-off in general. In addition, exponential cut-offs have been reported to become less pronounced in spatial-temporal measures of neurons that were acquired using imaging (Tagliazucchi et al., 2012; Ponce-Alvarez et al., 2018). This work is also spatial-temporal and thus could also be affected in such a manner. Thus, we *hypothesise* that the resulting nature of all three of these factors (using astrocytes rather than neurons, imaging under calcium fluorescence rather than long-term electrical recordings and spatial-temporal measurements rather than temporal measurements) would present a lower likelihood to observe an exponential cut-off.

Previous criticality analysis performed on astrocytes used a different definition of an avalanche, where instead of cells used in the formation of avalanches the pixels of the image were as demonstrated in criticality studies of astrocytes (Jung et al., 1998). This has the effect of combining both intracellular and intercellular Ca^2+^ waves into one data set. Using this definition, resulted in a larger number of avalanches being recorded as individual cells can create avalanches, whereas, in this analysis more than one cell was required. Under the definitions using pixels a shorter recording time (50 s) could be used while still producing sufficient data for the analysis. More recent studies of criticality in systems requiring Ca^2+^ imaging, such as beta cells, and cardiac myocytes employ the avalanche definition used in this work (Nivala et al., 2012; Gosak et al., 2017). Additionally, in the previous work on astrocyte network criticality only a size distribution with an exponent of 2.2 ± 0.2 was reported (Jung et al., 1998), compared to 2.06 ± 0.07 for the size distribution, and 2.80 ± 0.19 for the duration distribution calculated in this work. Other size exponents calculated for biological systems were 2.01 for whole brain recordings of zebra fish (using Ca^2+^ imaging), 1.92 for beta cells, and 1.5 for a neurons both in dissociated brain slices and neuronal cultures from a variety of species (Beggs and Plenz, 2003; Mazzoni et al., 2007; Pasquale et al., 2008; Klaus et al., 2011; Friedman et al., 2012; Hahn et al., 2017; Ponce-Alvarez et al., 2018). This variation could be explained by the differing definitions of an avalanche depending on the data acquisition method. Neuronal data are generally recorded from via electrodes and so an avalanche is a series of subsequent activations of electrodes. Models which capture critical dynamics have also been used to calculate the expected exponent value for certain universality classes. The most common example of these is the mean field size exponent of 1.5 which has been derived analytically (Sethna et al., 1993).

Mean field theory also predicts a duration distribution exponent of 2, which is found also in electrode recordings of neurons (Beggs and Plenz, 2003; Friedman et al., 2012; Ponce-Alvarez et al., 2018). However, simply calculating distribution exponents of similar value is thought to not be enough evidence to claim a system is at a critical point (Beggs and Timme, 2012; Hesse and Gross, 2014). This is because all systems of the same universality class should have the same exponents as well as the same universal function, which in the mean field theory is a perfectly inverted parabola (Sethna et al., 2006). In the case of human hNT astrocyte avalanches, the universal function is not a parabola as it rises to the maximum faster than it decreases, and so mean field theory may not be the universality class that best describes the type of criticality found in these cells. Astrocytes being of a different universality class to neurons is not unexpected as they rely on different communication pathways. Notably astrocytes display communication intracellularly, via gap junctions, and extracellularly through ATP neurotransmitter release. Implications of this are that one astrocyte may release neurotransmitter that activated neighbouring as well as distant cells. Extracellular stimulation may also elicit a different cellular response than intracellular stimulation, whereas neurons will always undergo an action potential when stimulated.

When a critical system is subject to an external driving force that is time dependent, the predicted values of the critical exponents change. In the case of a zero temperature random field Ising model this moves the size distribution exponent from 1.60 to 2.03 in a 3D model (Perković et al., 1999). In these experiments, the astrocytes imaged were embedded in a larger network of astrocytes which could not be imaged. Cells in this larger network could be thought of as an external driving force which may alter in time as they undergo Ca^2+^ waves. The size distribution exponent found in networks of hNT astrocytes is 2.06 which is in agreement with the zero temperature random field Ising model. In criticality research, the Ising model is typically used as a comparison rather than a definitive solution as it provides striking similarities between brain dynamics and the dynamics emerging from the Ising model at a critical temperature (Fraiman et al., 2009).

Shape collapse in the temporal profiles of avalanches is stronger evidence of a systems criticality than power-law exponents, as power-laws can be found in non-critical data (Stumpf and Porter, 2012). This has not been shown in networks of astrocytes previously. The ability to describe avalanche evolution across all size and duration scales using a universal function is a key component of critical networks (Sethna et al., 2001, 2006). While power-law exponents are regularly calculated for biological data finding shape collapse is less common. Universal functions found through shape collapse have been found in multiple types neuronal data. Cultured cortical slices of rat which were recorded from using electrode array where found to display shape collapse in avalanches in two out of ten cultures (Friedman et al., 2012). Shape collapse was also found in live imaging of zebra fish, using Ca^2+^, neuronal data (Ponce-Alvarez et al., 2018). More recent work using implanted microelectrodes in macaque monkeys found avalanches with signature parabolic profiles that could be collapsed to a single shape, and that filtration of certain neuron oscillations eliminated the signature relationships found in critical systems (Miller et al., 2019). Suggesting that neuron oscillations are embedded within avalanches found in networks of neurons (Gireesh and Dietmar, 2008; Lombardi et al., 2014). Additionally shape collapse is found in a wide variety of simulated models and non-biological experiments (Sethna et al., 2006).

Another aspect of criticality is the agreement of the exponents in the exponent relations, represented by the value of q , and closeness of b  and $\frac{1}{\sigma \nu z}$ . All networks have values of q that are close to unity. However the agreement between b  and $\frac{1}{\sigma \nu z}$ varies across networks.

The only criticality analysis that has been performed on cultures of human astrocytes, and that is most comparable to this work is the work by (Jung et al., 1998). Jung *et al* used astrocytes cultured from human epileptic foci, whereas this work has employed the hNT astrocyte which was not epileptic. Additionally, (Jung et al., 1998) recorded the cultured cells for 50 s, using images of 10,000 pixels. This work extended on these recordings and used image sequences of 40 min, with images of over 300,000 pixels. Another area where this work builds on (Jung et al., 1998) is in the fitting a validation of the power-law fit. Robust methods for analysing this type of distribution were not used until much later, and as such are missing from the work of (Jung et al., 1998).

Demonstrating that networks of human hNT astrocytes can display aspects of criticality is important as critical systems provide certain benefits, especially for system that process information (Muñoz, 2018). These include the dynamical range of the system’s ability to respond to a stimuli, as a supercritical system will respond to all stimuli by undergoing a response spanning all elements in the network, and a subcritical system can not produce a large response. Information transmission over the network is also enhanced, as well as long range correlations allowing coordination across the entire network (Shew and Plenz, 2013). Networks of neurons *in vitro*, in cortex slices, and *in vivo* demonstrated these benefits whereby the networks ability to respond to a range of stimuli and its ability to store and transmit information where reduced as the network was tuned, through application of drugs, further away from the critical point (Shew et al., 2009, 2011). Many of these benefits are thought to be broken down in certain diseases with epileptic networks being a model that has been studied (Hobbs et al., 2010; Meisel et al., 2012).

A wide variety of evoked and non-evoked astrocytic calcium waves have been observed *in vitro*, *in situ*, and *in vivo* experimental work. Non-evoked Ca^2+^ waves have been observed *in vitro*, *in vivo*, and *in situ* for astrocytes. *In vitro*, cultured primary mouse astrocytes have be observed to exhibit distinct oscillatory Ca^2+^ waves with durations ∼30 s (Lee et al., 2014) waves of approximately the same duration have also been previously observed in human hNT astrocytes (Hill et al., 2012). *In vivo*, imaging of cortical astrocytes in rats were found to display oscillatory Ca^2+^ waves with slow rise times separated by plateaus of durations ∼19 s (Nimmerjahn et al., 2004), other rat *in vivo* studies identified similar Ca^2+^ waves, which were termed as spikes, with an average duration of 25 s (Hirase et al., 2004). Similar Ca^2+^ waves were also observed in rat hippocampal astrocytes (Kuga et al., 2011). *In situ*, Ca^2+^ waves of astrocytes from rat ventrobasal thalamus slices have been reported with a half-maximal duration of 15 s (corresponding to durations ∼30–40 s), (Parri et al., 2001). Evoked Ca^2+^ waves of astrocytes *in vitro*, *in vivo*, and *in situ* have also been studied with distinct forms of Ca^2+^ waves occurring in response to different stimuli (Khakh and McCarthy, 2015). *In vitro*, ATP, glutamate and mechanical stimulation of primary rat astrocytes have been observed to evoke oscillatory Ca^2+^ waves with durations ∼ 7–12 s which were found to increase over in time after stimulus to between 30 and 45 s (Cornell-Bell and Fink, 1990; Charles et al., 1991; Guthrie et al., 1999). These same stimuli were found to elicit similar responses in human brain slices with ATP and glutamate evoked Ca^2+^ waves being between 20–30 s in duration (Oberheim et al., 2009). *In vivo*, localised Ca^2+^ waves with durations in the range of 9–36 s were reported in mouse cortical astrocytes after evoking whisker stimulation (Wang et al., 2006). Startling as well as adrenergic agonist application has also been show to evoke wider spread Ca^2+^ signalling with durations in the range of 10–20 s depending on the stimulus (Ding et al., 2013). It should be noted that whilst these studies have only been concerned with the mechanisms of calcium wave signal generation/propagation the Ca^2+^ wave duration is similar to the range that we observe here in human hNT astrocytes. This is interesting in itself as these studies span *in vitro*, *in vivo*, *in situ*, for evoked, non-evoked and intracellular and extracellular Ca^2+^ waves. Unfortunately, these studies have not been concerned with criticality and have thus not measured avalanche behaviour in order to estimate critical exponents. However, whilst only three criticality studies, including this one, have been performed in astrocytes (Jung et al., 1998; Wu et al., 2014) they span from rodents to this first study in humans. Thus, it is certainly plausible to hypothesis that avalanches most likely exist in complicated animal networks, but it is not possible to draw conclusions presently about criticality in these studies without the necessary measurements being made first. Thus, criticality studies could be used to help explain this apparent diversity within the models in the future.

It should be noted, that the astrocyte networks in this study were not tuned to criticality through the manipulation of a parameter, meaning that these networks are likely self-organized critical networks (Bak et al., 1988). Self-organization of astrocytes is possible as the cells are grown and allowed to network during the time between cell seeding and imaging (48 h). Self-organization is also thought to be the mechanism by which networks of neurons reach a critical state, thus, self-organization may play an important role in how the brain processes information in both the neuronal and astrocyte networks.

## Conclusion

In this work we recorded avalanches of Ca^2+^ waves in cultures of hNT astrocytes. These avalanches displayed aspects of critical dynamics, including power-law scaling in the duration and size distributions of avalanches, with exponents of 2.80 and 2.06 respectively. Shape collapse was shown in the temporal profiles of avalanches, with an exponent of 1.54. Other than a brief examination of the duration distribution in networks of astrocytes and the distributions in single cells, this is the first work that demonstrates characteristics of critical systems in human hNT astrocyte networks. Acknowledging that astrocytes are non-electrical cells and hence their activity must be studied using fluorescent imaging which leads to limitations in the duration of the experiment to 40 min. Our results show that astrocyte networks display characteristics of critical systems which is noteworthy, implying that astrocytes play a role in information processing alongside the well-established neural networks of the brain.

## Data Availability

The raw data supporting the conclusion of this article will be made available by the authors, without undue reservation.
